# Integrative Taxonomy Reveals *Molicola uncinatus* and *Gymnorhynchus gigas* (Cestoda: Trypanorhyncha) Coinfection in the Atlantic Pomfret *Brama brama* From the Mediterranean Sea, With Notes on the Phylogenetic Position of *G. gigas* Within the Family Gymnorhynchidae

**DOI:** 10.3389/fvets.2022.909163

**Published:** 2022-06-17

**Authors:** Mario Santoro, Marialetizia Palomba, Renato Aco Alburqueque, Simonetta Mattiucci

**Affiliations:** ^1^Department of Integrative Marine Ecology, Stazione Zoologica Anton Dohrn, Naples, Italy; ^2^Department of Biological and Ecological Sciences, University of Tuscia, Viterbo, Italy; ^3^Department of Public Health and Infectious Diseases, Section of Parasitology, Sapienza University of Rome, Rome, Italy

**Keywords:** larval trypanorhynchs, phylogenetic analysis, scanning electronic microscopy, 18S rDNA, 28S rDNA, fish parasites

## Abstract

The cestode family Gymnorhynchidae Dollfus, 1935 (Trypanorhyncha) comprises three genera and six valid species that are typically intestinal parasites of large pelagic sharks. Members of Gymnorhynchidae show a wide geographic distribution and represent a global sanitary concern because as larvae they infect the edible flesh of several commercially important fishes with some species having allergenic potential. Larval Gymnorhynchidae collected from the muscles of the Atlantic pomfret *Brama brama* from various localities in the Mediterranean Sea were identified and characterized by combining traditional morphology, scanning electronic microscopy, and molecular analyses using newly generated nuclear 18S and 28S rRNA sequences. Overall, 98 larvae were collected from 20 (100%) Atlantic pomfrets (intensity of infection: 4.9; range: 1–12). High-quality sequences were obtained for 54 larvae. Of these, 11 and 43 larvae were identified as *Molicola uncinatus* and *Gymnorhynchus gigas*, respectively. The phylogenetic analysis showed the existence of three main clades within Gymnorhynchidae. The first included species of *G. gigas* and *M. uncinatus* from the Mediterranean and Atlantic; the second and third major clades included an unidentified species of *Molicola* from the Indian Ocean and specimens of *Gymnorhynchus isuri* from the Mediterranean and Atlantic, respectively. Finally, *Chimaerarhynchus rougetae* was the basal and most diverging taxon. The phylogenetic analysis suggested that *G. gigas* is more closely related to the members of *Molicola*. We demonstrated the coinfection of *M. uncinatus* and *G. gigas* from all localities studied and extended the intermediate hosts and geographical range of *M. uncinatus* by including the Atlantic pomfret and the Tyrrhenian and Ionian Seas, respectively. The present results supported the previous proposal that *G. gigas* and *Molicola* species should be included in the same genus. Our study demonstrated the usefulness of the integrative taxonomy for the unequivocal recognition of larval trypanorhynch species, resolving the current difficulties in the taxonomy, and elucidating the poorly known ecological and biological aspects of members of Gymnorhynchidae.

## Introduction

The cestode family Gymnorhynchidae Dollfus, 1935 (order Trypanorhyncha) comprises three genera: *Gymnorhynchus* Rudolphi, 1819, *Molicola* Dollfus, 1935 and *Chimaerarhynchus* Beveridge and Campbell, 1989, with six valid species [i.e., *M. horridus* (Goodsir, 1841) Dollfus, 1935, *M. uncinatus* (Linton, 1924) Palm, 2004, *M. walteri* Palm, 2004, *G. gigas* (Cuvier, 1817) Rudolphi, 1819, *G. isuri* Robinson, 1959, and *C. rougetae* Beveridge and Campbell, 1989] (1–4). All species at the adult stage infect the intestine of large pelagic sharks (2, 4). The life cycle for members of this family has not yet been elucidated, but it has been proposed that copepods serve as first, pelagic euphausiids or schooling fish as second, and larger predatory fishes as third intermediate hosts (2). Species of Gymnorhynchidae show a wide geographic distribution and represent a global sanitary concern because as larvae they infect the edible flesh of several commercially important fishes (2).

According to the published records of Gymnorhynchidae in the Mediterranean Sea based on morphological identification of larvae, only *M. horridus* in the muscles of the swordfish *Xiphias gladius* (5, 6), and in the liver of the ocean sunfish *Mola* spp. (6–8), and *G. gigas* in the muscle of the Atlantic pomfret *Brama brama* [syn. *Brama raji*; (9, 10)] and of the silver scabbardfish *Lepidopus caudatus* (11–13) have been reported. Giarratana et al. (10) describing the infection of larvae of *G. gigas* in the muscles of the Atlantic pomfret from Sicilia and Calabria waters of southern Italy wrote “*Non si procedeva ad identificazione microscopica delle larve, considerata la nota, assoluta, prevalenza di Gymnorhynchus spp. in B. raji nell'ambito dei macroparassiti muscolari*.” The same method for larvae identification was used when Panebianco et al. (12) reported that larvae of *G. gigas* may interfere with values of total volatile basic nitrogen and trimethylamine nitrogen in the edible musculature of the silver scabbardfish. In both cases, Giarratana et al. (10) and Panebianco et al. (12) assumed that those larvae belonged to a single parasite species without even studying them. Recently, the application of molecular analyses for the identification of trypanorhynchs in Mediterranean fishes revealed that the larvae from the liver and muscles of the sunfish and the silver scabbardfish belonged to *G. isuri* (14) and *M. uncinatus* (15), respectively. This likely implies that the majority of the studies carried out so far from the Mediterranean Sea using questionable assumptions or morphological criteria alone for larvae identification reported misidentification of some Gymnorhynchidae species.

Nevertheless, misidentification of Gymnorhynchidae larvae using morphological criteria alone may occur because the isolation of their scoleces is difficult and often the partial or total invagination of their tentacles prevent the study of their most important taxonomic characteristics (2, 3, 14, 15). When this occurs, the use of molecular analysis allows for species identification of trypanorhynch larvae that are challenging or not possible (14–17). Correct identification of species is required to assess the biodiversity of an ecosystem. Misidentification of Gymnorhynchidae species can be confusing to our understanding of their ecological and biological aspects that include their poor known life cycles, geographical distribution, and host specificity (2). Moreover, regarding the sanitary risks for consumers, the correct identification of Gymnorhynchidae larvae from the flesh of fishes used for human consumption is of pivotal importance because of the allergenic potential of some species, such as *M. horridus* and *G. gigas* (18–21).

The aim of this study was to characterize, for the first time, by using an integrative taxonomic approach [scanning electronic microscopy (SEM) and molecular analysis], the larvae of Gymnorhynchidae that infect the edible musculature of the Atlantic pomfret in the Mediterranean and by generating new molecular data to discuss the phylogenetic relationships within the family.

## Materials and Methods

### General Data and Parasitological Analysis

**Figure 1:**
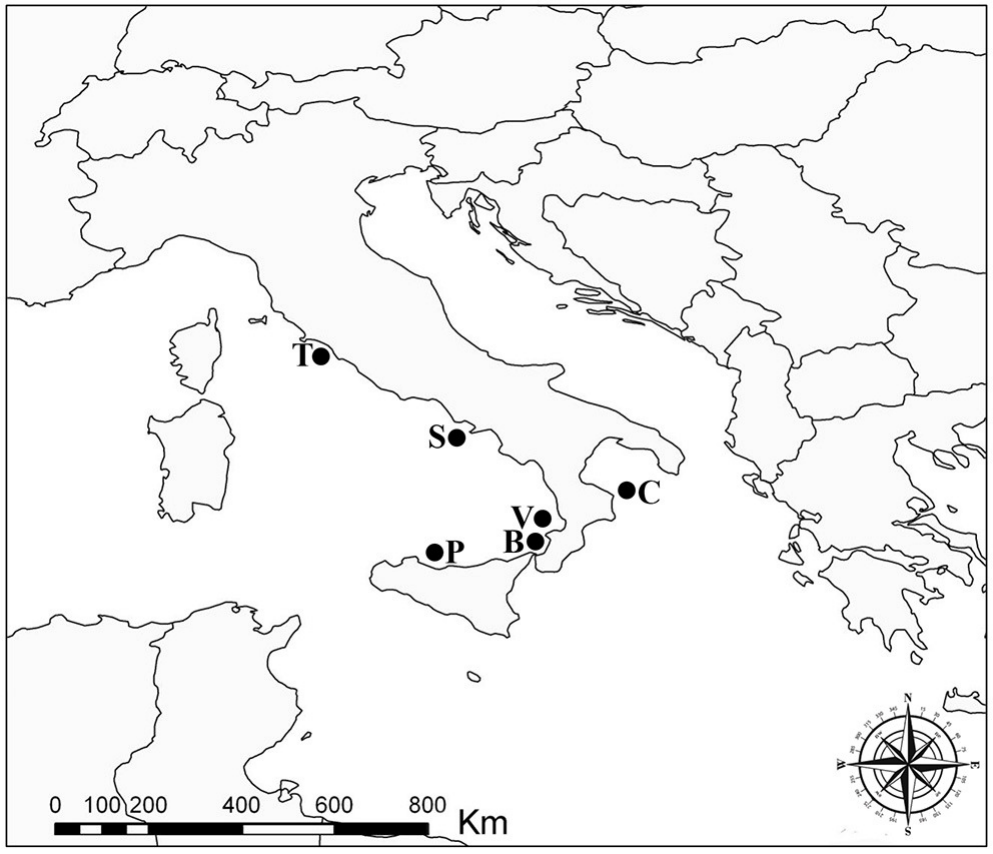
Sampling localities of the Atlantic pomfret in the Mediterranean Sea, 2021–2022. B, Bagnara Calabra; C, Cariati; P, Gulf of Palermo; S, Sorrento; T, Saline di Tarquinia; V, Vibo Valentia.

In total, 20 individuals of Atlantic pomfret were studied for trypanorhynch larvae between May 2021 and March 2022. Fish were obtained from off the Tyrrhenian coasts of Campania (Sorrento, *n* = 1), Latium (Saline di Tarquinia, *n* = 2), Sicily (Gulf of Palermo, *n* = 4), and Tyrrhenian and Ionian coasts of Calabria (Vibo Valentia, *n* = 3; Bagnara Calabra, *n* = 4; Cariati, *n* = 6) regions of Italy (Figure 1). Fish were collected by professional fishermen at ≈500 m depth using a longline and purchased on landing. The fish were refrigerated (+4°C) (*n* = 6) and examined within 24 h from capture or frozen (−20°C) (*n* = 14) for 2 weeks before thawing and dissection. They were measured (fork length—FL) to the nearest 0.1 cm and sex was determined by gonadal examination at dissection.

**Figure 2:**
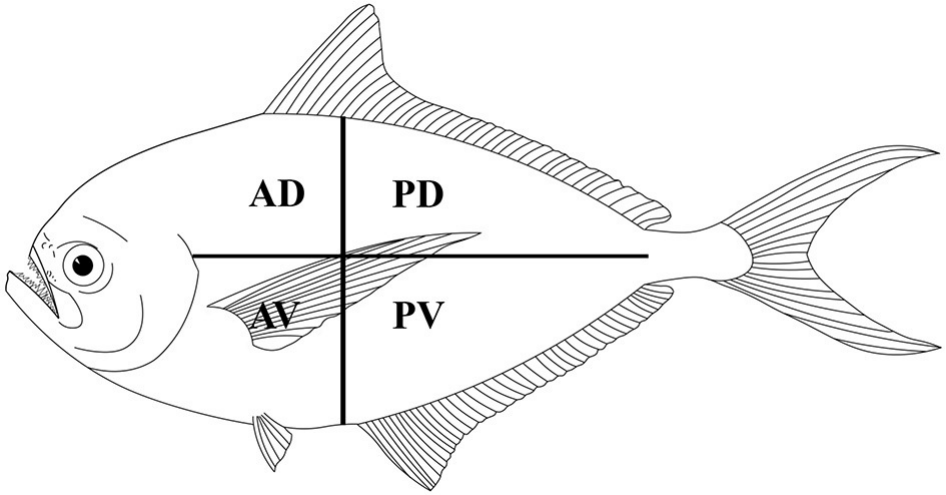
Schematic distribution of Gymnorhynchidae larvae in four muscle sections of the Atlantic pomfret. AD, anterior-dorsal; PD, posterior-dorsal; AV, anterior-ventral; PV, posterior-ventral.

Fish were manually eviscerated, and to assess the distribution of larvae in the skeletal muscles, the fillets of each fish were divided into four portions (i.e., anterior-dorsal, posterior-dorsal, anterior-ventral, and posterior-ventral; see Figure 2). Each portion of the fillets was first cut into thin slices and examined under a dissecting microscope for trypanorhynch larvae. Then, each slice was shredded in a Petri dish with purified seawater and all parasites were collected and counted (17). Live parasites were extracted from the muscles using scissors and tweezers and subsequently, were placed in Petri dishes with physiological saline or ringer lactate solutions (2, 3). To make free the scolex of larvae from the surrounding cyst, we used the methods described by Palm (2), i.e., slicing lengthwise the part below the pars bulbosa of the larva then the anterior part of the scolex was gripped and evaginated using forceps. The freed scoleces were placed overnight in saline or ringer lactate solutions to allow for evagination of their tentacles (2, 3).

Before preserving the scoleces in 70% ethanol for morphological identification, a fragment from the posterior portion of each scolex was frozen (−20°C) for molecular analyses. For morphological identification, the scoleces were clarified in Amman's lactophenol, and the main characters of the scolex and their tentacular apparatus were used to identify the larvae at the lowest possible taxonomic level, according to the identification keys proposed by Palm (2). The intensity of infection [±standard deviation] followed Bush et al. (22).

For SEM analyses, 10 scoleces were also fixed overnight in 2.5% glutaraldehyde, then transferred to 40% ethanol (10 min), rinsed in 0.1 M cacodylate buffer, postfixed in 1% OsO_4_ for 2 h, and dehydrated in ethanol series, critical point dried, and sputter-coated with platinum. Observations were made using a JEOL JSM 6700F SEM operating at 5.0 kV (JEOL, Basiglio, Italy). Parasites were identified at the species level according to the description of Palm (2) and Casado et al. (23, 24).

### Molecular and Phylogenetic Analyses

Total genomic DNA (gDNA) was extracted from 60 Gymnorhynchidae larvae, using the Quick-gDNA Miniprep Kit (Zymo Research), following the standard manufacturer-recommended protocol. The partial large subunit ribosomal DNA (lsrDNA, 28S) and the small subunit ribosomal DNA (ssrDNA, 18S) were amplified using the primers ZX-1 (5′-ACCCGCTGAATTTAAGCATAT-3′), 1500R (5′-GCTATCCTGAGGGAAACTTCG-3′) (25, 26), WormA (5′-GCGAATGGCTCATTAAATCAG-3′), and WormB (5′-CTTGTTACGACTTTTACTTCC-3′) (27), respectively.

Both polymerase chain reactions (PCRs) were carried out in a 25 μl volume containing 0.6 μl of each primer 10 μM, 2 μl of MgCl2 25 mM (Promega), 5 μl of 5 × buffer (Promega), 0.6 μl of deoxynucleoside triphosphates (dNTPs) 10 μM (Promega), 0.2 μl of Go-Taq Polymerase (5 U/μl) (Promega), and 2 μl of total DNA. PCR temperature conditions were the following: 94°C for 3 min (initial denaturation), followed by 35 cycles at 94°C for 30 s (denaturation), 53°C for 30 s (annealing), 72°C for 2 min (extension), followed by post-amplification at 72°C for 7 min. PCR amplicons were purified using the AMPure XP Kit (Beckman Coulter) following the standard manufacturer-recommended protocol and Sanger sequenced from both strands using the same primers through an Automated Capillary Electrophoresis Sequencer 3730 DNA Analyzer (Applied Biosystems), by the BigDye^®^ Terminator v3.1 Cycle Sequencing Kit (Life Technologies). Contiguous sequences were assembled and edited using MEGA X v. 11 (28). Sequence identity was checked using the Nucleotide Basic Local Alignment Search Tool (BLASTn) (29). The 28S and 18S sequences obtained in the present study were aligned with the available sequences of Gymnorhynchidae using ClustalX v. 2.1 (30). Regions of ambiguous alignment were defined in a NEXUS character exclusion set. The 28S and 18S sequences were combined, using SequenceMatrix (31), while the best partition schemes and best-fit models of substitution were identified using Partition Finder (32) with the Akaike information criterion [AIC; (33)].

The phylogenetic trees were constructed using both the Bayesian inference (BI) and maximum likelihood (ML) method. BI analysis was performed by MrBayes, v. 3.2.7 (34), using the Markov Chain Monte Carlo (MCMC) algorithm, with four chains, 0.2 as the temperature of heated chains, nst = 6, rates = invgamma, ngammacat = 4 models of evolution, 5,000,000 generations, with a subsampling frequency of 500, and a burn-in fraction of 0.25. Posterior probabilities (PP) were estimated and used to assess support for each node. Values with a 0.90 PP were considered well supported. ML analysis was performed using IQ-TREE (35) with 1,000 ultrafast bootstrap replicates (UFboot). Clades were considered to have high nodal support if the ML bootstrap resampling is ≥95%. The phylogenetic trees were rooted using *Pintneriella musculicola* Yamaguti, 1934 (15). For the phylogenetic tree construction, only the species with both 18S and 28S sequences were selected. The 28S and 18S sequences from GenBank included in the phylogenetic tree are listed in Table 1. Genetic distances were computed using the Kimura 2-Parameters (K2P) model (38) with 1,000 bootstrap resampling, by MEGA 7.0 (28). Representative sequences obtained in the present study were deposited in GenBank under the accession numbers ON197557-66 for 28S and ON197567-76 for 18S.

**Table 1:** Species, life-history (L, larva; A, adult), host species, locality of collection, and GenBank accession numbers of 28S and 18S sequences of taxa of the family Gymnorhynchidae included in the phylogenetic analysis shown in Figure 5.

**Species**	**Life-history**	**Host species**	**Locality**	**28S**	**18S**	**References**
*Chimaerahynchus rougetae*	A	*Squalus megalops*	New Caledonia	DQ642744	DQ642906	(36)
*Gymnorhynchus gigas*	L	*Brama brama*	Tyrrhenian Sea	ON197557-61	ON197572-76	This study
*Gymnorhynchus isuri*	A	*Isurus oxyrinchus*	USA	DQ642747	DQ642909	(36)
	L	*Mola mola*	Tyrrhenian Sea	MT667258	MT667257	(14)
*Molicola uncinatus*	L	*Thyrsites atun*	Australia	DQ642746	DQ642908	(36)
	L	*Lepidopus caudatus*	Malta coast	MT823197	MT823193	(15)
		*Mola mola*	Tyrrhenian Sea	ON197562-66	ON197567-70	This study
*Molicola* sp.	L	*Taractes rubescens*	Indonesia	FJ572949	FJ572913	(25)
	L	*Xiphias gladius*	Sri Lanka	KX712337-41	KX712332-36	(37)
*Pintneriella musculicola* (outgroup)	A	*Odontaspis ferox*	Indonesia	FJ572948	FJ572912	(25)

## Results

### General Data and Parasitological Analysis

**Figure 3:**
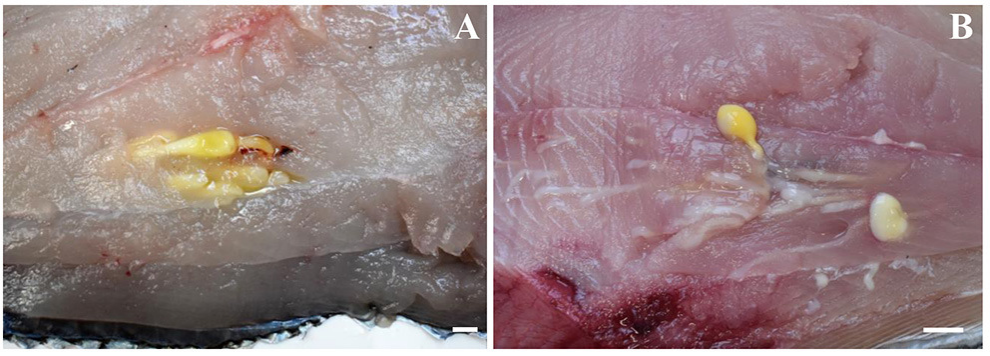
Larvae of *Molicola uncinatus* **(A)** and *Gymnorhynchus gigas* **(B)** sequenced in the present study from the muscles of the Atlantic pomfret.

**Figure 4:**
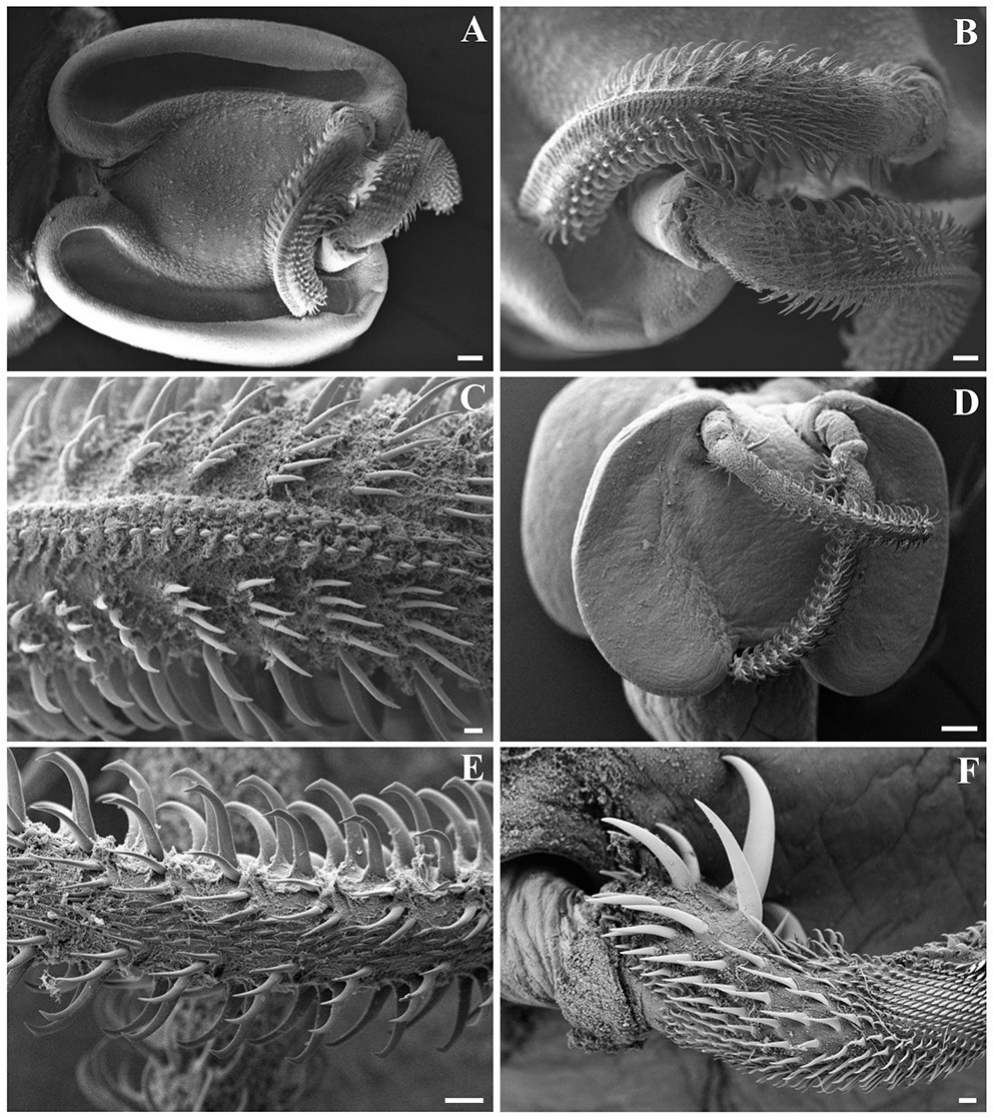
Scanning electron micrographs of *Gymnorhynchus gigas* **(A–C)** and *Molicola uncinatus* **(D–F)** sequenced in the present study. **(A,D)** Bothria and tentacular apparatus. **(B,C,E,F)** Particular of tentacles. Note the presence of winged chainette elements on the external surface of metabasal armature of *G. gigas* **(B,C)**, and the beaklike tips of principal hooks and the band of spiniform hooks on the external surface of metabasal armature of *M. uncinatus* **(E)**. **(F)** Basal armature of tentacle of *M. uncinatus*.

Out of the 20 Atlantic pomfret, nine were males and 11 were females ranging in FL from 26.5 to 47.5 cm. Overall 98 larvae were collected from 20 (100%) Atlantic pomfrets. The intensity of infection was 4.9 ± 3.2 ranging from 1 to 12. Most of the larvae were found in the posterior ventral portion (73.5%) of the fillets, the rest were from the posterior dorsal (14.3%) and anterior ventral portions (12.2%) of the fillets. Larvae from the skeletal muscles of the Atlantic pomfret were large and whitish or yellowish (Figure 3), with a sub-spherical blastocyst surrounding the invaginated scolex. Based on the morphological characters of the scolex, all larvae examined were conformed to earlier descriptions of genera of Gymnorhynchidae. We obtained only 18 scolices with at least a tentacle completely everted just from the refrigerated fish to be used for morphological study. These larvae based on the morphological characters were assigned to *M. uncinatus* (*n* = 6) and *G. gigas* (*n* = 12), respectively (Figure 4). In particular, the specimens of *M. uncinatus* showed a band of spiniform hooks on the external surface of the tentacle, uncinated hooks 1(1′) of similar size to hooks 2(2′), hooks 4–6(4′–6′) beaklike, and 12–15 macrohooks at the base of the tentacle (vs. 8–10 and 25–30 macrohooks in *M. horridus* and *M. walteri*, respectively). Conversely, the specimens of *G. gigas* showed a double chainette of elements on the middle of the external tentacle surface and more than 20 falciform macrohooks at the tentacle base (vs. 8–15 in *G. isuri*).

### Molecular and Phylogenetic Analyses

**Figure 5:**
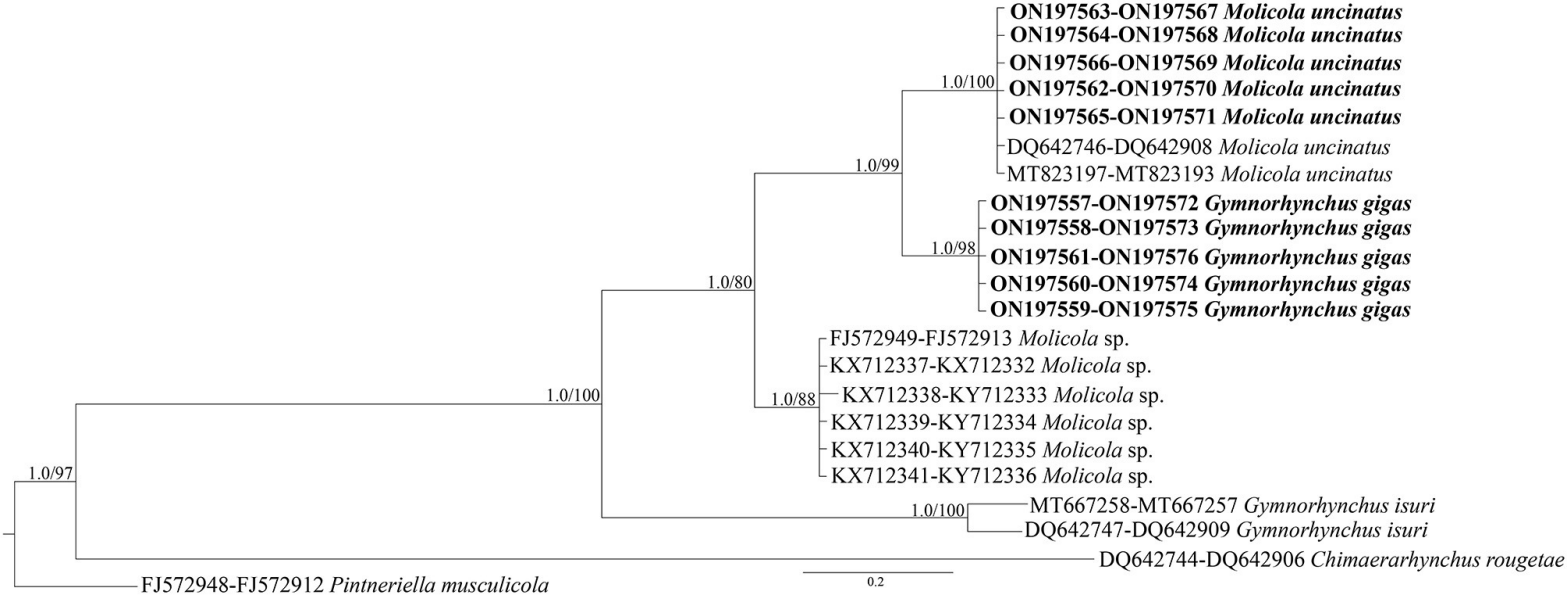
Phylogenetic concatenated tree from maximum likelihood (ML) based on 28S and 18S sequences obtained in the present study (in bold), with respect to the Gymnorhynchidae sequences at the same gene loci available in GenBank. Nodal support is indicated for BI (posterior probabilities) and ML (ultrafast bootstrap), respectively. *Pintneriella musculicola* was used as outgroup.

High-quality sequences at both 28S (1,450 bp) and 18S (1,928 bp) gene loci were successfully obtained for 54 larvae. According to BLAST analysis, 11 larvae showed 100% similarity with the sequences (28S: MT823197-DQ642746 and 18S: MT823193-DQ642908) of *M. uncinatus*, available in GenBank. The remaining sequences of 43 larvae obtained at the 28S and 18S gene loci, of which 12 individuals had been morphologically identified as *G. gigas*, showed ≈98.90 and 99.40% similarities, respectively, with the sequences of *M. uncinatus* (28S: MT823197-DQ642746 and 18S: MT823193-DQ642908) available in GenBank. Coinfection by both parasite species was found in all localities studied. Genetic distance values between species of Gymnorhynchidae based on 28S and 18S sequences data sets are given in Table 2. While no intraspecific variation was found, a certain degree of genetic differentiation was observed at the interspecific level. In particular, the K2P value ranged between *M. uncinatus* and *G. gigas* from a minimum of 0.0082 ± 0.0024 and 0.0039 ± 0.0023, to a maximum of 0.0791 ± 0.0080 and 0.0559 ± 0.0089 between *G. isuri* and *C. rougetae*, at the 28S and 18S gene loci, respectively. Phylogenetic tree was carried out using both separately (data not shown) and combined 18S and 28S gene loci data sets (Figure 5). Because BI and ML analyses yielded identical topologies, only ML is shown in Figure 5. Within the family Gymnorhynchidae, three clades were detected. Specimens of *M. uncinatus* and *G. gigas* were clustered in a first clade with high nodal support (PP = 1.0, UFboot = 99). In particular, the newly generated sequences of *M. uncinatus* clustered with high nodal support (PP = 1.0, UFboot = 100) with those of *M. uncinatus* previously deposited in GenBank. In the same clade, a novel sister clade of *M. uncinatus* was formed with high nodal support (PP = 1.0, UFboot = 100) by the newly generated sequences of *G. gigas*. The second major clade, with high nodal support (PP = 1.0, UFboot = 88), included an unidentified species of *Molicola*. The third clade was formed by specimens of *G. isuri*.

**Table 2:** K2P genetic distance ± standard error (SE) between species of family Gymnorhynchidae.

	***M. uncinatus***	***Molicola*** **sp**.	***G. gigas***	***G. isuri***	***C. rougetae***
*M. uncinatus*	-	0.0095 ± 0.0035	0.0039 ± 0.0023	0.0195 ± 0.0050	0.0522 ± 0.0082
*Molicola* sp.	0.0137 ± 0.0030	-	0.0055 ± 0.0027	0.0156 ± 0.0044	0.0497 ± 0.0081
*G. gigas*	0.0082 ± 0.0024	0.0156 ± 0.0033	-	0.0154 ± 0.0044	0.0495 ± 0.0080
*G. isuri*	0.0349 ± 0.0049	0.0279 ± 0.0046	0.0381 ± 0.0054	-	0.0559 ± 0.0089
*C. rougetae*	0.0744 ± 0.0078	0.0780 ± 0.0082	0.0771 ± 0.0080	0.0791 ± 0.0080	-

*28S K2P values are reported below the diagonal and 18S K2P values are above the diagonal*.

## Discussion

Trypanorhynchs have a unique morphological feature, with both adults and larvae having the scolex equipped with a tentacular apparatus consisting of four retractile tentacles adorned with hooks (2, 39). According to Palm (2), the three genera of Gymnorhynchidae can be distinguished on the basis of the armature patterns of their tentacles (heterocanthous typical in *Molicola*; poeciloacanthous multitypical in *Gymnorhynchus*; and poeciloacanthous typical in *Chimaerarhynchus*) and the absence (in *Molicola*) or presence (in *Gymnorhynchus* and *Chimaerarhynchus*) of winged chainette elements on the surface of their tentacles. Morphological characters of the present specimens of *M. uncinatus* and *G. gigas* were in agreement with descriptions of Palm (2) and Casado et al. (23, 24). Molecular analysis confirmed the morphological identification of *M. uncinatus* being the sequences obtained identical to those of *M. uncinatus* previously collected in *Thyrsites atun* and *L. caudatus* (15, 36). The sequences of *G. gigas* here obtained, revealing closer similarity with those of *M. uncinatus*, represent the first molecular data available for *G. gigas*, which should be considered a starting point for comparative analyses and molecular identification of this species. In the present study, the identification of Gymnorhynchidae larvae found in the musculature of the Atlantic pomfret from various localities within the Mediterranean was obtained by combining traditional morphology, SEM, and molecular analyses because only a few live larvae everted their tentacles, which represent organs with essential diagnostic characters (2, 39). Therefore, the use of the integrative taxonomy allowed for accurate discrimination between *M. uncinatus* and *G. gigas*.

The supposed life-history strategy of Gymnorhynchydae might explain the coinfection with *M. uncinatus* and *G. gigas* in the Atlantic pomfret. According to the parasite-host list of Palm (2) and subsequent literature (15, 36), *M. uncinatus* infects *Alopias vulpinus* (Lamniformes) as definitive host and *Thyrsites atun, Taractes rubescens, Taractichthys steindachneri*, and *L. caudatus* (Scombriformes) as intermediate hosts. However, as a sporadic finding, it was also reported in the liver of *M. mola* (Tetraodontiformes), and in the musculature of *Allothunnus fallai* (Scombriformes) and *X. gladius* (Carangiformes) (2). Conversely, *G. gigas* infects *Carcharodon carcharias, Isurus oxyrinchus, Lamna nasus* (Lamniformes), and *Oxynotus centrina* (Squaliformes) as definitive hosts and *B. brama* as intermediate host (2). The present results confirm the importance of Scombriformes fishes in the life-cycle of both the trypanorhynch species. Moreover, all Scombriformes usually involved in their biological cycle are benthopelagic fishes (occurring along the upper slope down to 400–1,000 m), which prey on small fishes, cephalopods, amphipods, and euphausiids (40). It has been suggested that different species of trypanorhynchs have evolved different life-history strategies occupying different ecological niches, also in terms of vertical distribution (2, 17, 41). Indeed, each parasite species has its depth preferences, following the most common feeding ecology and depth range of its definitive host. Currently, there is no information on the feeding behavior of the Atlantic pomfret, however, studies on the feeding ecology of other Bramidae fishes and known intermediate and definitive hosts of *G. gigas* and *M. uncinatus* support that these fishes take consistent vertical movements in the water column for feeding (40). The vertical migration could allow the infection with Gymnorhynchidae by feeding on infected prey, and at the same time, it could increase the possibility of parasite transmission exposing the Atlantic pomfret to the attack of pelagic sharks. Our results suggest that the benthopelagic deep habitat may represent the preferred food-web system in which larvae of *M. uncinatus* and *G. gigas* are found in the Mediterranean.

The high migratory habits of the known intermediate and definitive hosts of both *M. uncinatus* and *G. gigas* confirm that their wide geographical dispersion may be associated with the movements of their hosts (2, 42). Indeed, the geographical range so far reported of *M. uncinatus* and *G. gigas* seems to mirror the migratory behavior and distribution of their definitive hosts being both known from north and south-east Pacific, north-west and south Atlantic, North Sea, and Mediterranean Sea (2, 15). The Atlantic pomfret and the Tyrrhenian and Ionian Seas represent new host and new geographical records for *M. uncinatus*, respectively. Indeed, in the Mediterranean, this parasite has only been recently found off the Malta coast (15).

Regarding the larvae distribution, the higher occurrence of both species in the ventral portions of fillets may be linked to the shortest distance of the larvae penetrating from the guts into the musculature as proposed by Seyda (43), while the higher occurrence in its posterior portions could provide greater possibilities to enhance the transmission of parasites. This is in agreement with previous observations (16, 17, 44) and may be explained by the hunting strategy followed by several sharks (definitive hosts), which use a tail-on approach, preying from behind.

Phylogeny of Gymnorhynchidae derived from the sequences available in GenBank plus those generated in the present study revealed the existence of three main clades. The first involved species of *G. gigas* and *M. uncinatus* all from Mediterranean teleosts, except for a specimen of *M. uncinatus* collected from the south-east Pacific (Australian waters) in *T. atun* (36). The second and third clades included an undetermined species of *Molicola* infecting the swordfish *X. gladius*, the pomfret *Taractes rubescens*, and *Taractichthys steindachneri* from the Indian Ocean, and *G. isuri* infecting *M. mola* and *I. oxyrinchus* from the Mediterranean and Atlantic, respectively. Finally, *C. rougetae* was the basal and most diverging taxon in all the elaborations from the two different data sets.

The phylogenetic analysis based on both genetic markers supported the hypothesis that *G. gigas* is very closely related to members of the genus *Molicola* rather than to the other species of the genus *Gymnorhynchus*. This finding seems to suggest that the morphological character, i.e., the presence vs. the absence of winged chainette elements on the external surface of their tentacles as proposed by Palm (2) to differentiate *Gymnorhynchus* and *Molicola*, respectively, has no taxonomic value for the genus diagnosis. A similar situation was found in the phylogenetic inference of the anisakid species of the *Contracaecum osculatum* (*s.l*.) (Rudolphi, 1802) Baylis, 1920 complex plus *Phocascaris cystophorae* (Berland, 1963), which formed a well-supported monophyletic group (46), in spite of the morphological character presence/absence of interlabia on which the distinctiveness at the genus level, has been based. Unfortunately, the anatomical characters of segments of adult *G. gigas* type species of the genus *Gymnorhynchus* are unknown as this species was described from larval material (2, 3, 6, 45). This prevents the identification of further distinctive morphological characters between the two genera until a redescription of adult *G. gigas* from the type locality will be made.

The present phylogenetic results support Dollfus's (6) proposal according to which *G. gigas* and *Molicola* species were included in the same genus. Indeed, Dollfus (6) placed *Gymnorhynchus* and *Molicola* in the same genus suggesting the subgenus *Molicola* for species that did have a band of hooks on the external surface of their tentacles instead of winged chainette elements. Afterward, based on the tentacle characters, the members of *Gymnorhynchus* were separated into the families Gymnorhynchidae and Molicolidae by Beveridge and Campbell (1). A subsequent cladistic analysis based on shared morphological characters of 49 recognized genera of Trypanorhyncha supported the close relationship between Gymnorhynchidae and Molicolidae (45), then more recently, Palm (2) included *Gymnorhynchus* and *Molicola* into the same family.

## Conclusions

The present study demonstrated the usefulness of the integrative taxonomy for the unequivocal recognition of larval trypanorhynch species in fish when coinfection with similar species occurs. The present phylogenetic analysis also strongly supported the hypothesis that *G. gigas* is very closely related to species of *Molicola*. Finally, the coinfection within the musculature of the same fish species from various Mediterranean localities and the strict phylogenetic relationships between *G. gigas* and *M. uncinatus* could suggest a similar coevolutionary history that could have accompanied the speciation of these cestodes in their definitive hosts. Nevertheless, additional molecular and new co-phylogenetic data could clarify the patterns of host-parasite coevolutionary events between these parasites and their hosts.

## Data Availability Statement

The datasets presented in this study can be found in online repositories. The names of the repository/repositories and accession number(s) can be found in the article.

## Ethics Statement

Ethical review and approval was not required for the animal study because the fish used in the present study were purchased dead on landing and no permit and/or ethical consideration were needed.

## Author Contributions

MS, SM, and MP conceived and designed the study. MS, MP, and RA performed fish parasitological analysis, parasites collection, morphological and molecular analysis, and data elaboration. MS wrote the manuscript. All authors read, reviewed, and approved the final manuscript.

## Funding

This study was performed in the frame of the research “Food-web transmitted endoparasites and their hosts: an integrative approach to investigate the “state” of biodiversity of the marine ecosystem from off Calabria coast” carried out under the project “Centro Ricerche ed Infrastrutture Marine Avanzate in Calabria (CRIMAC)”, funded by FSC 2014-2020 - Piano Stralcio Ricerca e Innovazione 2015–2017 - Programma Nazionale Infrastrutture di Ricerca (PNIR), linea d'azione 1. Cofinanziamento Infrastrutture di Ricerca (IR).

## Conflict of Interest

The authors declare that the research was conducted in the absence of any commercial or financial relationships that could be construed as a potential conflict of interest.

## Publisher's Note

All claims expressed in this article are solely those of the authors and do not necessarily represent those of their affiliated organizations, or those of the publisher, the editors and the reviewers. Any product that may be evaluated in this article, or claim that may be made by its manufacturer, is not guaranteed or endorsed by the publisher.

## References

[1] Beveridge I Campbell RA. *Chimaerarhynchus* n. g. and Patellobothrium n. g. two new genera of trypanorhynch cestodes with unique poeciloacanthous armatures, and a reorganisation of the poeciloacanthous trypanorhynch families. Syst Parasitol. (1989) 14:209–25. 10.1007/BF02187055

[2] Palm HW. The Trypanorhyncha Diesing 1863. Bogor: PKSPL-IPB Press (2004). p. 710.

[3] Robinson ES. Some new cestodes from New Zealand marine fishes. Trans Proc R Soc N Z. (1959) 86:381–92.

[4] Global Cestode Database. Tapeworm Species Search. (2022). Available online at: https://tapewormdb.uconn.edu/ (2022) (accessed April 12, 2022).

[5] Manfredi MT Traldi G Gandini G. Cestoda larvae of Trypanorhyncha in muscles of broadbill swordfishes (*Xiphias gladius*). Atti della Societa Italiana delle Scienze Veterinarie. (1993) 47:765–7.

[6] Dollfus RP. Etudes critiques sur les Tétrarhynques du Muséum de Paris. Arch Mus Hist Nat. (1942) 19:1–466.

[7] Gustinelli A Nardini G Aureli G Trentini M Affronte M Fioravanti ML. Parasitofauna of *Mola mola* (Linnaeus, 1758) from Italian seas. Biol Mar Med. (2006) 13:872–6.

[8] Ahuir-Baraja AE Yamanoue Y Kubicek L. First confirmed record of *Mola* sp. A in the western Mediterranean Sea: morphological, molecular and parasitological findings. J Fish Biol. (2017) 90:1133–41. 10.1111/jfb.13247 28105658

[9] Panebianco F. Brama raji (BI.). Biologia, caratteri morfologici e strutturali, metodi di pesca, parassitosi, linea di condotta nella i. c. Il Progresso Veterinario. (1952) 8:277–81.

[10] Giarratana F Muscolino D Ferrara P Panebianco A. Accertamenti ispettivi approfonditi sul pesce castagna (*Brama raji*). Ital J Food Saf. (2012) 1:30–5. 10.4081/ijfs.2012.6.30

[11] Giuffrida A Pennisi L Bottari T Panebianco A. Indagine sull'attività proteolitica di *Gymnorhynchus gigas* e della muscolatura infestata di *Lepidopus caudatus*. Atti Associazione Italiana Veterinari Igienisti. (2002) 12:305−6.

[12] Panebianco A Signorino D Muscolino D Giarratana F. Larve plerocercoidi di *Gymnorhynchus* (Cestoda: Trypanorhyncha) in *Lepidopus caudatus*: interferenza nella determinazione di TMA-N e AVBT. Ital J Food Saf. (2011) 1:77–9. 10.4081/ijfs.2011.1.77

[13] Giarratana F Muscolino D Beninati C Ziino G Giuffrida A Trapani M. *Gymnorhynchus gigas* in *Lepidopus caudatus* (Actinopterygii: Perciformes: Trichiuridae): prevalence and related effects on fish quality. Czech J Food Sci. (2014) 32:320–5. 10.17221/330/2013-CJFS

[14] Santoro M Palomba M Mattiucci S Osca D Crocetta F. New parasite records for the sunfish *Mola mola* in the Mediterranean Sea and their potential use as biological tags for long-distance host migration. Front Vet Sci. (2020) 7:579728. 10.3389/fvets.2020.579728 33195589 PMC7641614

[15] Palomba M Santoro M Aco Alburqueque R Cipriani P Mattiucci S. First molecular detection of the parasites *Molicola uncinatus* and *Hepatoxylon trichiuri* (Cestoda: Trypanorhyncha) infecting the silver scabbardfish *Lepidopus caudatus* from the Central Mediterranean Sea: implications for the seafood quality and safety. Food Control. (2021) 122:107807. 10.1016/j.foodcont.2020.107807

[16] Santoro M Degli Uberti B Corrado F Cutarelli A Iaccarino D Di Nocera F. *Grillotia* (Cestoda: Trypanorhyncha) plerocerci in an anglerfish (*Lophius piscatorius*) from the Tyrrhenian Sea. Parasitol Res. (2018) 117:3653–58. 10.1007/s00436-018-6067-4 30178197

[17] Santoro M Bellisario B Crocetta F Degli Uberti B Palomba M. A molecular and ecological study of *Grillotia* (Cestoda: Trypanorhyncha) larval infection in small to mid-sized benthonic sharks in the Gulf of Naples, Mediterranean Sea. Ecol Evol. (2021) 11:13744–55. 10.1002/ece3.7933 34707814 PMC8525172

[18] Vazquez-Lopez C De Armas-Serra C Bernardina W Rodriguez-Caabeiro F. Oral inoculation with *Gymnorhynchus gigas* induces anti-parasite anapyhylactic antibody production in both mice and rats and adverse reactions in challenge mice. Int J Food Microbiol. (2001) 64:307–15. 10.1016/S0168-1605(00)00477-3 11294352

[19] Vazquez-Lopez C De Armas-Serra C Bernardina W Rodriguez-Caabeiro F. A. 24-kDa collagenase from *Gymnorhynchus gigas* elicits rat ileum hyperreactivity and is a target of humoral responses in mice previously given a single oral dose of parasite extract. Dig Dis Sci. (2002) 47:935–42. 10.1023/a:1014785210407 11991631

[20] Gòmez-Morales MA Ludovisi A Giuffra E Manfredi MT Piccolo G Pozio E. Allergenic activity of *Molicola horridus* (Cestoda, Trypanorhyncha), a cosmopolitan fish parasite, in a mouse model. Vet Parasitol. (2008) 157:314–20. 10.1016/j.vetpar.2008.07.010 18790571

[21] Pelayo V García-Hernández P Puente P Rodero M Cuébllar C. Seroprevalence of anti-*Gymnorhynchus gigas* (Trypanorhyncha, Gymnorhynchidae) antibodies in a Spanish population. J Parasitol. (2009) 95:778–80. 10.1645/GE-1894.1 19206995

[22] Bush AO Lafferty KD Lotz JM Shostak AW. Parasitology meets ecology on its own terms: Margolis et al. revisited. J Parasitol. (1997) 83:575–83. 10.2307/3284227 9267395

[23] Casado N Moreno M Urrea-París M Rodriguez-Caabeiro F. Ultrastructural study of the papillae and presumed sensory receptors in the scolex of the Gymnorhynchus gigas plerocercoid (Cestoda: Trypanorhyncha). Parasitol Res. (1999) 85:964–73. 10.1007/s004360050667 10599918

[24] Casado N Urrea MA Moreno MJ Rodriguez-Caabeiro F. Tegumental topography of the plerocercoid of *Gymnorhynchus gigas* (Cestoda: Trypanorhyncha). Parasitol Res. (1999) 85:124–30. 10.1007/s004360050520 9934961

[25] Palm HW Waeschenbach A Olson PD Littlewood DTJ. Molecular phylogeny and evolution of the Trypanorhyncha Diesing, 1863 (Platyhelminthes: Cestoda). Mol Phylogenet Evol. (2009) 52:351–67. 10.1016/j.ympev.2009.01.019 19489123

[26] Van der Auwera G Chapelle S De Wachter R. Structure of the large ribosomal subunit RNA of *Phytophthora megasperma*, and phylogeny of the oomycetes. FEBS Lett. (1994) 338:133–6. 10.1016/0014-5793(94)80350-1 8307170

[27] Littlewood DTJ Olson PD. Small subunit rDNA and the Platyhelminthes: Signal, noise, conflict and compromise. In: Littlewood DTJ, Bray RA, editors. Interrelationships of the Platyhelminthes. London: Taylor & Francis (2001). p. 262–78.

[28] Kumar S Stecher G Li M Knyaz C Tamura K. MEGA X: molecular evolutionary genetics analysis across computing platforms. Mol Biol Evol. (2018) 35:1547–9. 10.1093/molbev/msy096 29722887 PMC5967553

[29] Morgulis A Coulouris G Raytselis Y Madden TL Agarwala R Schaffer AA. Database indexing for production MegaBLAST searches. Bioinformatics. (2008) 24:1757–64. 10.1093/bioinformatics/btn322 18567917 PMC2696921

[30] Larkin MA Blackshields G Brown NP Chenna R McGettigan PA McWilliam H. Clustal W and clustal X version 20. Bioinformatics. (2007) 23:2947–8. 10.1093/bioinformatics/btm404 17846036

[31] Vaidya G Lohman DJ Meier R. SequenceMatrix: concatenation software for the fast of multi-gene datasets with character set and codon information. Cladistics. (2011) 27:171–80. 10.1111/j.1096-0031.2010.00329.x 34875773

[32] Lanfear R Calcott B Ho SYW Guindon S. PartitionFinder: combined selection of partitioning schemes and substitution models for phylogenetic analyses. Mol Biol Evol. (2012) 29:1695–701. 10.1093/molbev/mss020 22319168

[33] Akaike H. Information theory and an extension of the maximum likelihood principle. In: Petrov BN, Csaki F, editors. International Symposium on Information Theory. Budapest, HUNG: Akademiai Kiado (1973). p. 267–81.

[34] Huelsenbeck JP Ronquist F. MRBAYES: Bayesian inference of phylogeny. Bioinformatics. (2001) 17:754–5. 10.1093/bioinformatics/17.8.754 11524383

[35] Nguyen LT Schmidt HA von Haeseler A Minh BQ. IQ-TREE: a fast and effective stochastic algorithm for estimating maximum-likelihood phylogenies. Mol Biol Evol. (2015) 32:268–74. 10.1093/molbev/msu300 25371430 PMC4271533

[36] Olson PD Caira JN Jensen K Overstreet RM Palm HW Beveridge I. Evolution of the trypanorhynch tapeworms: parasite phylogeny supports independent lineages of sharks and rays. Int J Parasitol. (2010) 40:223–42. 10.1016/j.ijpara.2009.07.012 19761769

[37] De Silva D Perera J Fernando H Ranatunga R De Silva B. Molecular identification of the genus *Molicola* larvae from swordfish (*Xiphias gladius*) captured in Sri Lanka by ribosomal subunit gene sequencing. JAFH. (2021) 10:66–74. 10.20473/jafh.v10i1.20905

[38] Kimura M. A simple method for estimating evolutionary rates of base substitutions through comparative studies of nucleotide sequences. J Mol Evol. (1980) 16:111–20. 10.1007/BF01731581 7463489

[39] Campbell RA Beveridge I. Order Trypanorhyncha diesing, 1863. In: Khalil LF, Jones A, Bray RA, editors. Keys to the Cestode Parasites of Vertebrates. Wallingford: Commonwealth Agricultural Bureaux International (1994). p. 51148.

[40] Froese R Pauly D. FishBase. Brama Bloch & Schneider 1801 (2022). Available online at: https://www.marinespecies.org/aphia.php?p=taxdetails&id=125924 (accessed February 8, 2022).

[41] Palm HW Yulianto I Piatkowski U. Trypanorhynch assemblages indicate ecological and phylogenetical attributes of their elasmobranch final hosts. Fishes. (2017) 2:8. 10.3390/fishes2020008

[42] Palm HW Waeschenbach A Littlewood DT. Genetic diversity in the trypanorhynch cestode *Tentacularia coryphaenae* Bosc, 1797: evidence for a cosmopolitan distribution and low host specificity in the teleost intermediate host. Parasitol Res. (2007) 101:153–9. 10.1007/s00436-006-0435-1 17216487

[43] Seyda M. On a case of mass invasion of cestode *Gymnorhynchus* (*Gymnorhynchus*) *gigas* (Cuvier, 1817) larvae in muscles of *Brama raii* (Bloch, 1791). Acta Ichthyol Piscat. (1976) 6:59–65. 10.3750/AIP1976.06.1.04

[44] Dallarés S. Twenty thousand parasites under the sea: a multidisciplinary approach to parasite communities of deep-dwelling fishes from the slopes of the Balearic Sea (NW Mediterranean). (dissertation/PhD Thesis). Universitat Autònoma de Barcelona, Barcelona, Spain (2016).

[45] Beveridge I Campbell RA Palm HW. Preliminary cladistic analysis of genera of the cestode order Trypanorhyncha Diesing, 1863. Syst Parasitol. (1999) 42:29–49. 10.1023/A:1006011512221 10613545

[46] Mattiucci S Paoletti M Webb SC Sardella N Timi JT Berland B. Genetic relationships among species of *Contracaecum* Railliet & Henry, 1912 and *Phocascaris* Höst, 1932 (Nematoda: Anisakidae) from pinnipeds inferred from mitochondrial *cox2* sequences, and congruence with allozyme data. Parasité. (2008) 15:408–19. 10.1051/parasite/2008153408 18814715

